# Supplementary figures and images for: Dopamine Receptor 1 Specific CRISPRa Mice Exhibit Disrupted Behaviors and Striatal Baseline Cellular Activity
Source: eNeuro. 2025 Aug 8;12(8):ENEURO.0157-25.2025. doi: 10.1523/ENEURO.0157-25.2025 (PMC12360625; doi:10.1523/ENEURO.0157-25.2025)

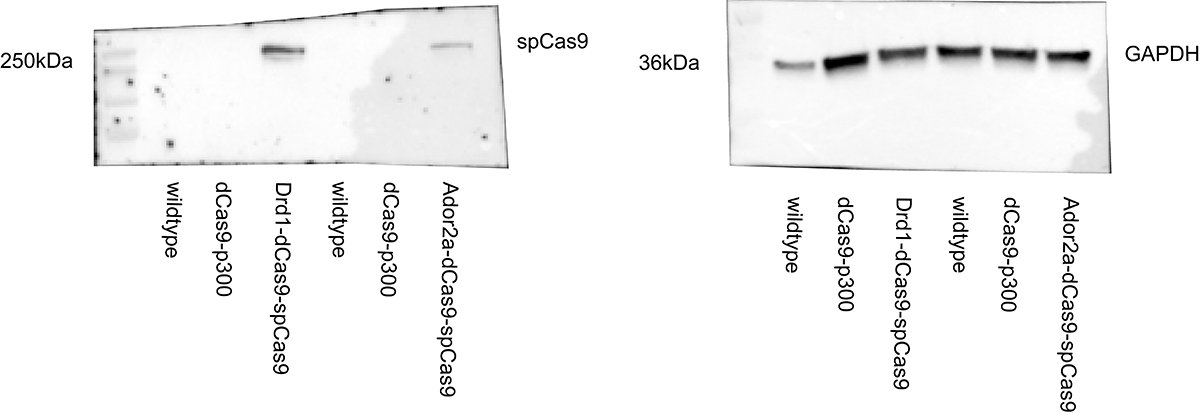

Supplement: Figure 1-1 — Full Western blot data of CRISPRi mice for Cas9 expression validation. Samples in order from left to right are: wildtype, dCas9-p300, Drd1-Cre:dCas9-p300, wildtype, dCas9-p300 and Adora2a-Cre:dCas9-p300. The blot was cut at the 50 kDa ladder mark. On the left is the half of the blot that was >50 kDa which was probed with an Anti-Cas9 antibody. Cas9 was detected near the 250 kDa marker in only the Drd1-Cre:dCas9-p300 and Adora2a-Cre:dCas9-p300 samples. On the right is the half of the blot that was <50 kDa, which was probed with an Anti-GAPDH antibody. GAPDH was detected in all samples. Download Figure 1-1, TIF file. [file eneuro-12-ENEURO.0157-25.2025-s001.tif]

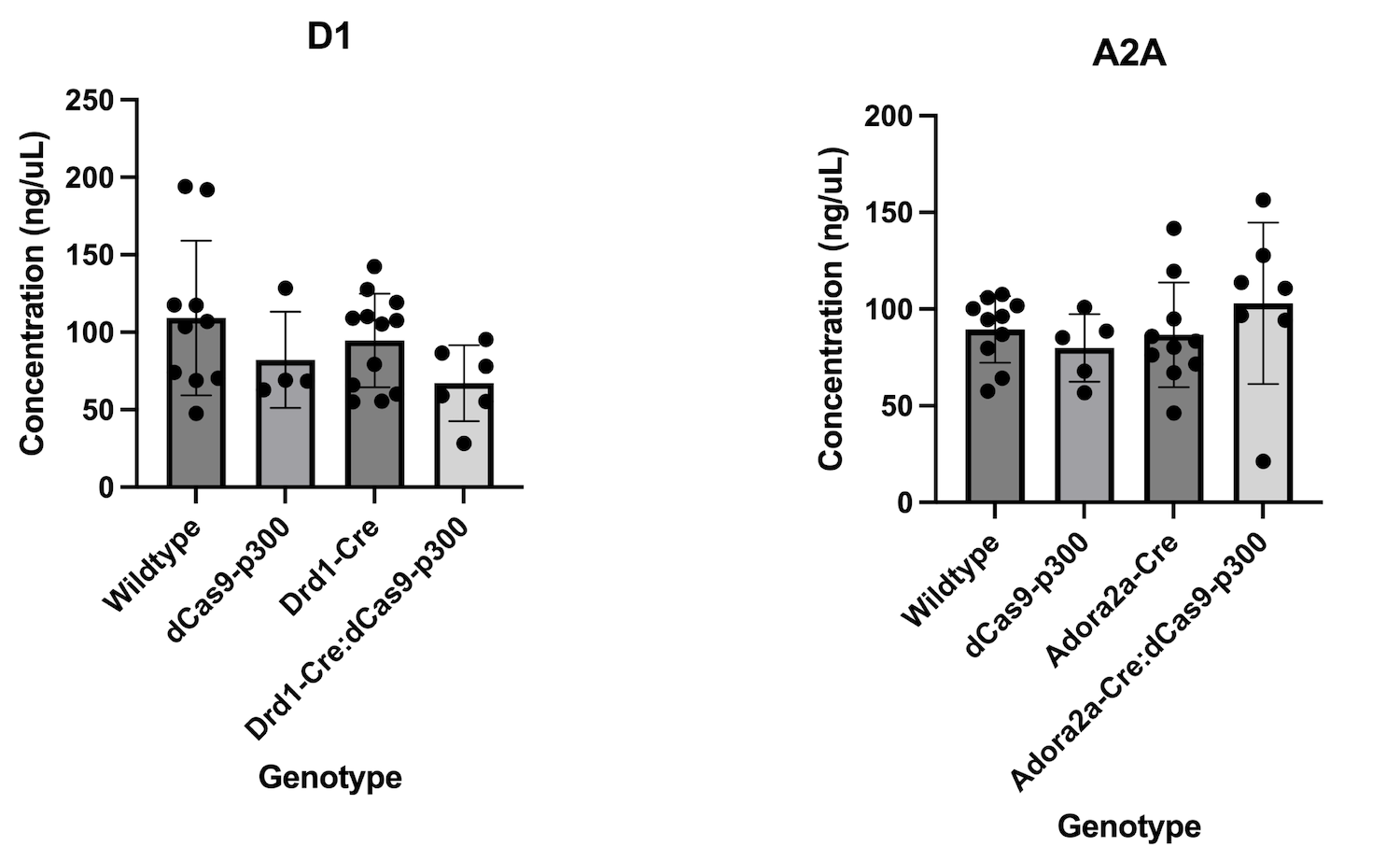

Supplement: Figure 2-1 — RNA concentration (in ng/µL) from NAc tissue from mice. There was no significant difference in RNA yield between the genotypes for each line. Download Figure 2-1, TIF file. [file eneuro-12-ENEURO.0157-25.2025-s003.tif]
