# Supplementary material for: Dopamine Receptor 1 Specific CRISPRa Mice Exhibit Disrupted Behaviors and Striatal Baseline Cellular Activity
Source: eNeuro. 2025 Aug 8;12(8):ENEURO.0157-25.2025. doi: 10.1523/ENEURO.0157-25.2025 (PMC12360625; doi:10.1523/ENEURO.0157-25.2025)
Supplement: Figure 1-2 — RNAscope quantification. Download Figure 1-2, DOCX file. [file eneuro-12-ENEURO.0157-25.2025-s002.docx]

Figure 1-2. RNAscope quantification.
